# Open reduction and locked compression plate fixation, with or without allograft strut, for periprosthetic fractures in patients who had a well-fixed femoral stem: a retrospective study with an average 2-year follow-up

**DOI:** 10.1186/s12891-022-05008-2

**Published:** 2022-01-19

**Authors:** Hui Lv, Xing Guo, Yuan Hui Wang, Zhong Jie Zhang, Long Fei Zou, Hao Xue, Deng Hua Huang, Mei Yun Tan

**Affiliations:** Department of Orthopedic Surgery, Affiliated Hospital of Southwest Medical University, No. 25 Taiping Road, Luzhou, 646000 Sichuan China

**Keywords:** Periprosthetic fractures, Vancouver, Cortical strut allograft, Locking compression plate, Open reduction and internal fixation

## Abstract

**Background:**

The use of cortical strut allograft has not been determined for Vancouver type B1 or C fracture. This study aimed to evaluate the short-term efficacy of locking compression plating with or without cortical strut allograft in managing these types of fractures.

**Methods:**

We retrospectively assessed 32 patients (17 males, 15 females; 23–88 years, mean: 67.2 years) with Vancouver type B1 or C fractures. Seventeen patients (Group A; B1 fractures in 15 hips, C fractures in 2 hips) were treated with open reduction and internal fixation with locking compression plates (group A). The other 15 patients (Group B; B1 in 14 hips, C in 1 hip) were fixed by locking compression plating combined with cortical strut allografting (group B). The fracture healing rate, healing time, complications and function were compared between these two groups.

**Results:**

The mean follow-up time was 32.4 months (12 to 66), and the overall fracture union rate of the 32 patients was 96.9%. Group B had a higher fracture union rate than Group A, but the difference was not statistically significant. Group A had one case of nonunion of type B1 fracture and one case of malunion; the mean time to fracture healing was 5.3 months (3 to 9). In group B, all patients reached bony union without malunion, with a mean time of fracture healing of 5.1 months (3 to 8).

**Conclusion:**

Treatment of Vancouver type B1 or C fractures by locking compression plating, with or without cortical strut allografting, resulted in similar union rates in these patients. This suggest that, the use of cortical strut allografting should be decided cautiously.

## Introduction

The number of total hip arthroplasty (THA) operations has been growing globally, with increasing use in younger patients [1]. As a result, periprosthetic fractures are also becoming increasing common. A study form Mayo Clinic reported a 20-year incidence of periprosthetic fractures of 3.5% [2]. In 2013, a study of the French THA registry found periprosthetic fractures to be the second leading cause for THA revision (11.8%), only after aseptic loosening [3]. Challenges reported for managing these fractures include the proximal medullary cavity filled by the femoral stem, comminuted fracture, osteopenic bone associated with periprosthetic fracture often leading to fracture nonunion, refracture and poor function [4–6]. The choice of treatment method is often based on fracture classification. Currently, the Vancouver classification, proposed by Duncan and Masri in 1995, is the most widely used periprosthetic fracture classification [7]. The treatment of periprosthetic fractures is mainly divided into revision arthroplasty and open reduction and internal fixation (ORIF), which is considered as the first choice for Vancouver type B1 fracture. Locking plating is frequently selected in the operative management of Vancouver type B1 fracture [8], but the use of cortical strut allografts remains inconclusive [9, 10]. Such cortical strut has two major advantages that make it a useful complement to other methods of fixation devices: reconstruction of bone stock [8, 11] and improving the stability of fracture fixation [12]. Many studies have reported a higher union rate and it is recommended to routinely use cortical strut allograft for the treatment of periprosthetic fractures [13–15]. However, in recent years, studies have reported that locking plate alone can still achieve high union rate and function [16–18], and the use of cortical strut allografts also has disadvantages, such as high infection rates, soft tissue stripping and expensive material [19]. To date, there are only limited comparative studies on cortical strut allograft for Vancouver type B1 and C fracture. Therefore, this retrospective study aimed to compare the short-term efficacy of locking compression plating with or without cortical strut allograft, and to identify the indications of cortical strut allografting.

## Materials and methods

**Fig. 1:**
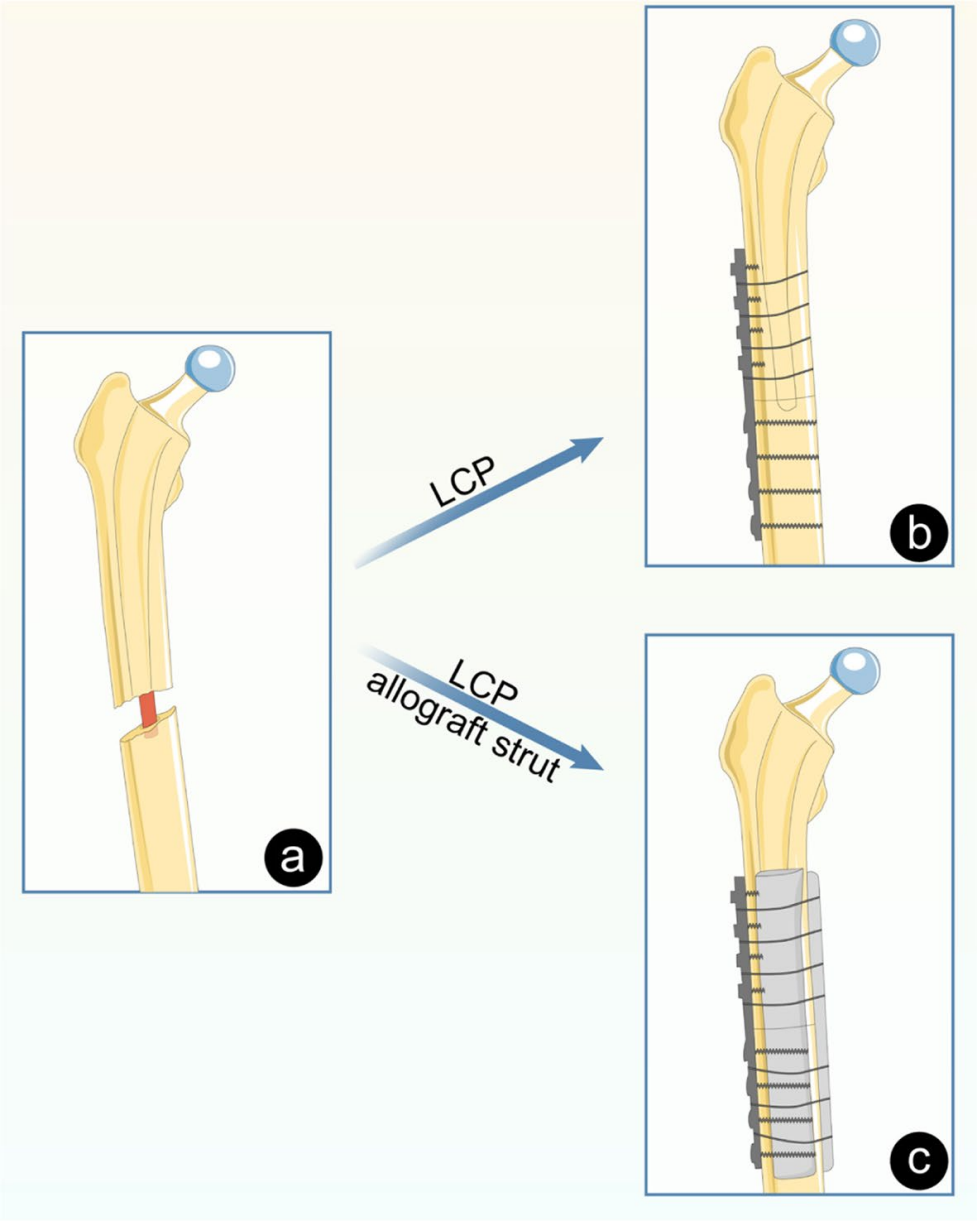
Periprosthesis fracture model and two different therapeutic plans. a Schematic diagram of type B1 Periprosthesis fracture. **b** Type B1 Periprosthesis fracture were fixed by locking compression plating. **c** Type B1 Periprosthesis fracture were fixed by locking compression plating combined with cortical strut allografting and the allograft strut located on the anterior and medial side of the femoral stem

This retrospective study was approved by the Ethics Committee of the Hospital, and all patients provided informed consents to be included in the study. We reviewed the clinical and radiographic data of all patients with periprosthetic fractures classified as Vancouver type B1 or C after THA between March 2010 and February 2019 registered in the electronic medical record system. A total of 39 patients entered in this study. Of these, 3 patients underwent revision arthroplasty, 2 chose conservative treatment, and 34 received locking compression plating with or without cortical strut allografts. Two patients were lost to follow-up as they migrated other provinces. Finally, 32 patients ((17 males, 15 females; 23–88 years, mean: 67.2 years) were included in this study. Of these, 30 patients experienced periprosthetic fractures after primary THA, and the other 2 after revision THA. The reasons for their primary THA were: avascular necrosis of the femoral head (13 hips, 40.6%), femoral neck fractures (12 hips, 37.5%), developmental dysplasia of the hip (4 hips, 12.5%), and other conditions (3 hips, 9.4%). The causes of revision THA were periprosthetic fracture (one patient) and aseptic loosening (one patient). The mean body mass index (BMI) was 22.8 kg/m^2^ (15.1 to 29.0 kg/m^2^). All patients had a history of trauma, mostly caused by low energy. Specifically, 29 patients (90.6%) fell from their standing height, and 3 (9.4%) slipped while climbing and falling stairs. There were 24 cases (75%) of uncemented femoral stem and 8 cases (25%) of cemented femoral stem. The mean time from primary hip arthroplasty to periprosthetic fracture was 4.9 years (0.05 to 27). The patients were divided into two groups (Table 1); Group A received no cortical strut allografting, whereas Group B received such treatment. A schematic diagram of the two different therapeutic plans was exhibited in Fig. 1.

**Table 1:** Patient demographics and Clinical characteristics

Variable	Group A LCP alone (*n* = 17)	Group B LCP and allografting (*n* = 15)	*p*-value
Age, mean (SD), yrs	65.2 (16.9)	69.4 (11.5)	0.435
**Sex, no./total no. (%)**			0.723
Male	10/17 (66.7%)	7/15 (60.0%)	
Female	7/17 (33.3%)	8/15 (40.0%)	
Course, mean (SD), yrs	4.2 (4.9)	5.7 (7.1)	0.692
BMI, mean (SD)	22.6 (3.1)384.2	23.1 (3.5)	0.666
**Reason for primary arthroplasty, no. (%)**			0.825
Femoral neck fracture	6/17 (36.8)	7/15 (40.0)	
Osteonecrosis of femoral head	6/17 (36.8)	6/15 (33.4)	
Developmental dysplasia of hip	3/17 (15.9)	1/15 (13.3)	
Other	2/17 (10.5)	1/15 (13.3)	
**PPFF type, no. (%)**			1.000
Vancouver type B1	15/17 (84.2)	14/15 (93.3)	
Vancouver type C	2/17 (15.8)	1/15 (6.7)	
**Femoral stem type, no. (%)**			0.423
Cemented	3/17 (21.0)	5/15 (33.4)	
Uncemented	14/17 (79.0)	10/15 (66.6)

All operations were performed by two senior orthopaedic surgeons. In all patients with type B1 fracture, the posterolateral extension approach was used. The incision length was ranging from 18 cm to 23 cm (mean: 20). First, the hip joint was dislocated through the posterolateral approach. The stability of the femoral stem was tested by longitudinal traction, and the rotational torque was measured following earlier studies [15, 20]. After confirming that femoral stem was stable, the fracture was exposed again via the posterolateral approach, and the fracture was reduced under direct vision and fixed by locking compression plating (LCP) (Waston, Changzhou, Jiangsu, China) with or without the combined use of fresh frozen cortical strut allografts. We strictly followed the technique described in previous studies for the implantation of LCP devices, with a minimum of 3 unicortical locked screws at the proximal end of the fracture and at least 2 bicortical locked distal screws, combined with cerclage wire (Fig. 1b). To secure the fixation, three patients in Group A were fixed with proximal femoral trochanter claw plate and inserting two locking screws at the greater trochanter. For cortical strut allografting, we performed the allograft on the anterior and medial side of the femoral stem, and four double-strand steel wires were girded for the first fixation (Fig. 1c). The length of the strut allograft was > 6 cm above and below the broken end of the fracture, which was implanted with morselized cancellous allograft. For type C fracture, direct lateral incision was used directly. Drainage tubes were placed in all patients and removed in 2 days postoperatively. There were 17 cases of LCP group (Group A), 15 cases of type B1 fracture and 2 cases of type C fracture, including 4 cases of 12-hole plate, 7 cases of 14-hole plate, 3 cases of 16-hole plate, 3 cases of femoral trochanter plate, 15 cases of LCP combined with cortical strut allograft (Group B), 14 cases of type B1 fracture and 1 case of type C fracture, including 4 cases of 12-hole plate, 6 cases of 14-hole plate and 5 cases of 16-hole plate (Table 2). Patients in both groups were fixed with at least 3 wires.

**Table 2:** Operative details for all patients

Variable	LCP(*n* = 17)	LCP and allograft (*n* = 15)	*p*-value
operative duration, mean (SD), mins	143.2 (41.8)	166.3 (54.8)	0.187
Blood loss, mean (SD), ml	770.5 (367.0)	860.0 (470.2)	0.551
**LCP type, no./total no. (%)**			0.452
12 hole	4/17 (26.3)	4/15 (26.7)	
14 hole	7/17 (36.9)	7/15 (40.0)	
16 hole	3/17 (15.8)	4/15 (20.0)	
Other	3/17 (21.0)	0/15 (13.3)	
**wire/cable, no./total no. (%)**			0.016
3 to 6	7/17 (36.9)	1/15 (13.3)	
6 to 8	8/17 (42.1)	6/15 (33.4)	
>8	2/17 (21.0)	8/15 (53.3)	

Postoperative prophylactic use of antibiotics and antithrombotic was routinely performed. All patients were encouraged to undergo quadriceps muscle and ankle pump exercise on the day of the surgery, and they began non-weight bearing walk with the assistance of walking aids or crutches on the second day after surgery. With the help of radiography, the affected limbs were evaluated and allowed to gradually walk with weight bearing. The follow-up times was 3, 6, 12 months after the operation and at least once each year. The patients who were not convenient to come to the hospital for follow-up were informed to gather the information by telephone interview, and the lateral femoral radiographs were taken at the local hospital and mailed to the follow-up staff. The Harris hip score (HHS) [21] was used to evaluate hip function. Anteroposterior and lateral radiographs of the femur on the operative side were taken to evaluate whether there was loosening of the prosthesis, migration of plate and screw, and fracture healing. Fracture healing was determined by clinical healing and radiological evidence. Corten et al. recommended that [20], conditions for clinical healing include full weight bearing (with or without assistive devices), no or only mild occasional pain, and no impact on walking or basic daily activities. Radiological evidence for fracture healing was bridging callus formation in both anteriorly and laterally of the femur. Malunion was defined as sagittal or medial deviation from the anatomical standard > by 5°.

Continuous variables between the two groups (e.g., HHS and age) were compared by t-test or Mann-Whitney U test (SPSS V.21.0, SPSS, Chicago, IL, USA). Dichotomous variables from 2 groups were analyzed by χ^2^ test or Fisher’s exact test. A *p*-value < 0.05 was considered statistically significant.

## Results

The mean follow-up time of 32 patients was 32.4 months (12 to 66). One patient died of pulmonary infection 15 months after surgery, and another died of cardiovascular disease 32 months after surgery. There was no statistical significance in preoperative age, BMI and fracture type between the two groups. Notably, the mean age of patients in Group B was greater than that in Group A and the difference was statistically significant.

**Fig. 2:**
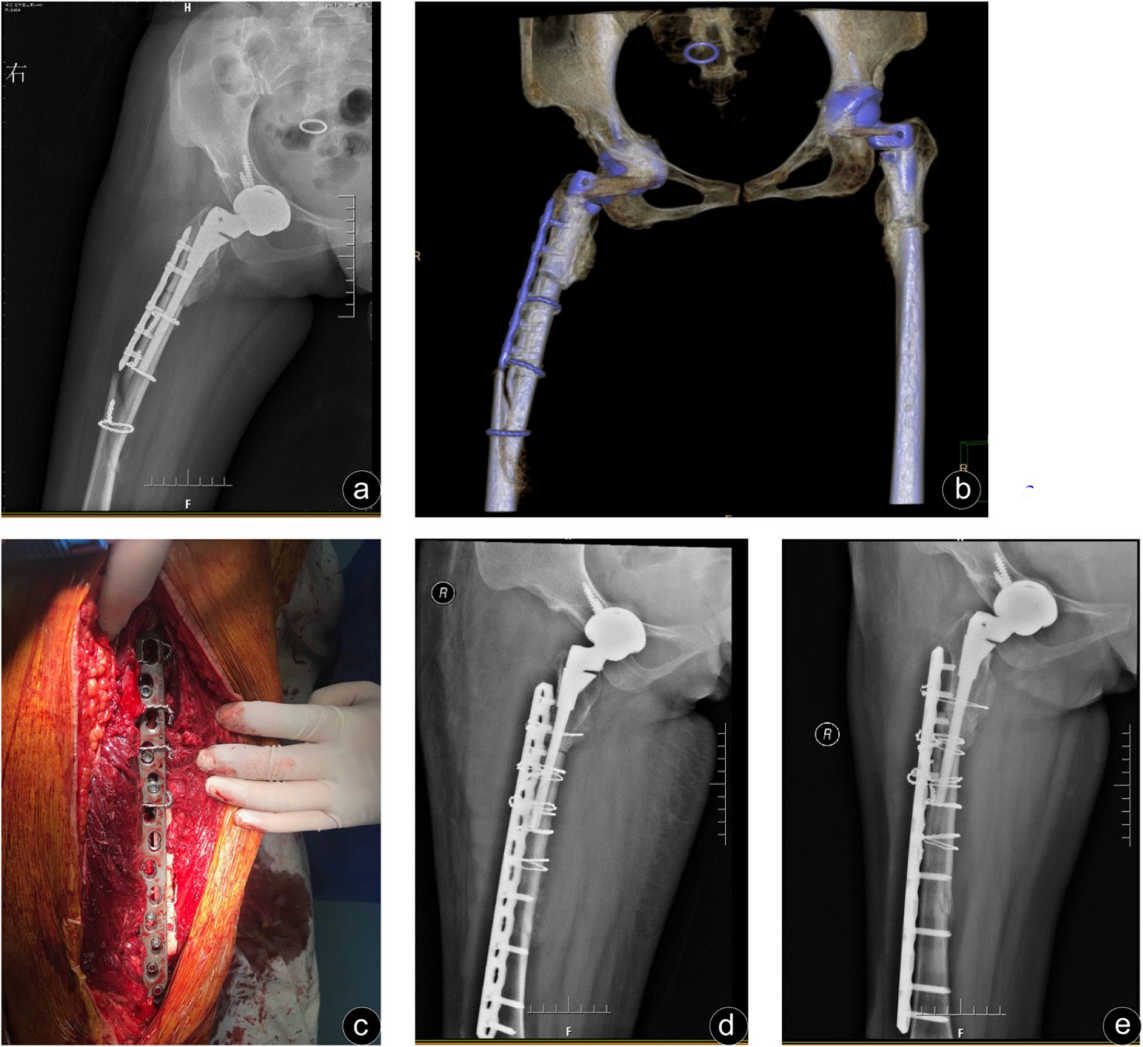
A 41-year-old female who sustained a Vancouver type B1 fracture 5 months after primary total hip arthroplasty due to Crowe type IV hip dysplasia. **a** and **b** Preoperative anteroposterior X-ray and three-dimensional reconstruction of the femur showed an oblique fracture of the distal right femoral stem. **c** Locked compression plate and encircling steel wire were used for fixation during operation. **d** The anteroposterior radiographs on the third day after the operation showed satisfactory fracture reduction, good alignment and normal pseudographs. **e** Anteroposterior radiographs 6 months after the operation showed distal union of the fracture, but malunion at the proximal osteotomy with a 15-degree varus deformity

**Fig. 3:**
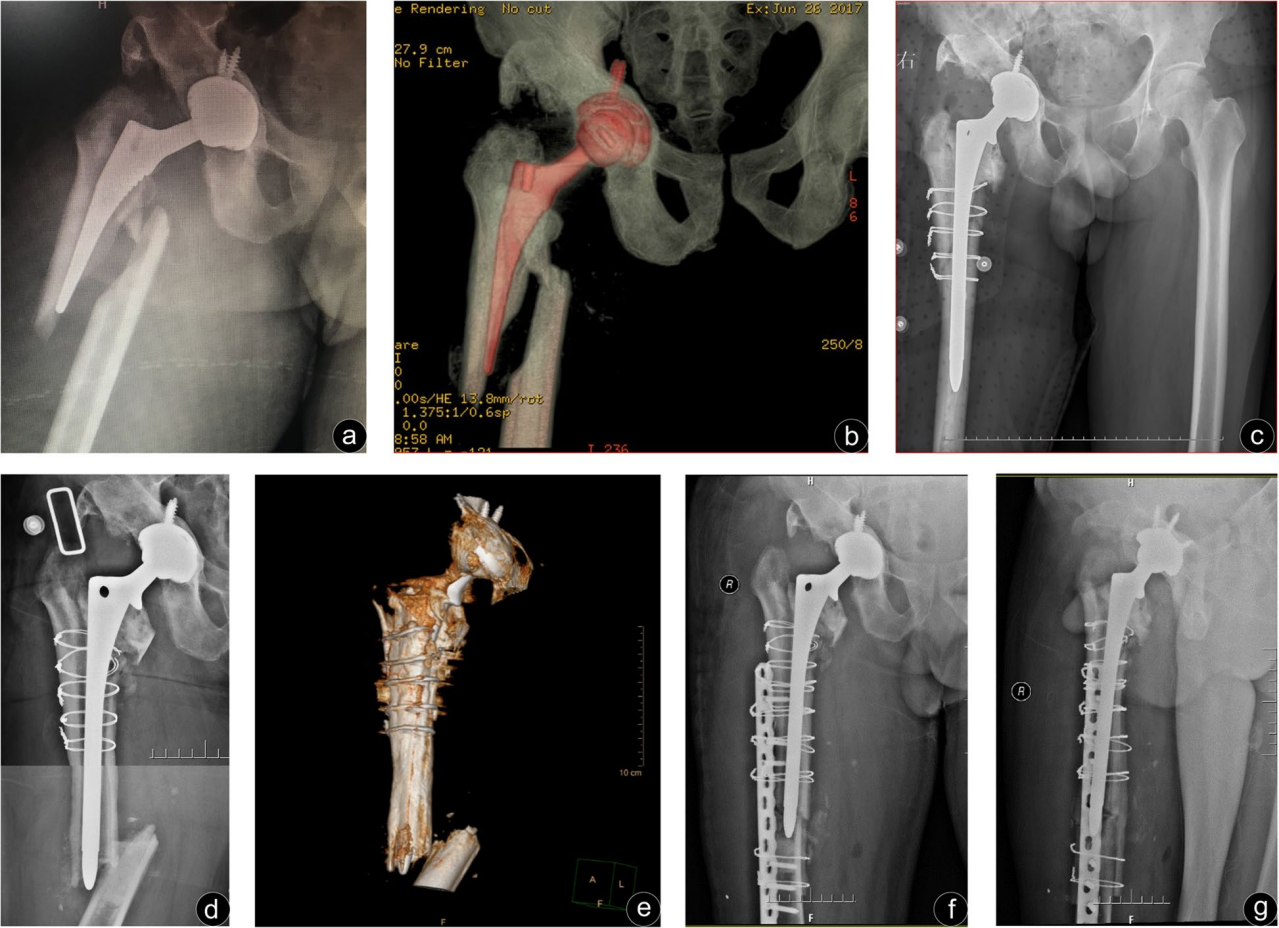
A 53-year-old male who sustained a periprosthetic femur fracture 2 years after primary total hip arthroplasty. Three years after revision, there was another periprosthetic fracture. **a**, **b** Preoperative anteroposterior X-ray and three-dimensional reconstruction of the femur showed a spiral fracture. No signs of prosthesis loosening were observed. **c** The femoral stem was loosened intraoperatively and long-stem revision was used for treatment. **d**, **e** The anteroposterior radiographs and three-dimensional reconstruction of the femur showed a short transverse fracture. **f**, **g** The anteroposterior radiographs on the 2 weeks after the operation which use locked compression plate Fixation, with allograft strut showed satisfactory fracture reduction and good alignment

The two groups had an overall fracture union rate of 96.9%. The fracture union rate of Group B was higher than that of Group A, but the difference was not statistically significant. In Group A, there were 1 case of nonunion of type B1 fracture and 1 case of malunion, and all of type C fractures reached bony union. The mean time to fracture healing was 5.3 months.

(3 to 9). The reasons responsible for nonunion and malunion in patients may be interpreted to be too short plate length and unstable proximal fixation. In Group B, all fractures were bony union without malunion, and the mean time of fracture healing was 5.1 months (3 to 8). Two typical cases are illustrated in Figs. 2 and 3.

In terms of postoperative function, the mean HHS of Group A was 78.1 (35–98) and Group B was 79.4 (48–96) at the last follow-up or at the end of the follow-up. There was no significant difference in HHS between the two groups (*p* = 0.807). The mean operation time of Group A was 143.2 min (100 to 230), and the mean blood loss was 770.6 ml (350 to 2000). The mean operation time of Group B was 166.3 min (110 to 270), and the blood loss was 860 ml (300 to 1500). The score measurement of the two groups at the last postoperative follow-up was shown in Table 3. There was no significant difference in HHS pain scores between the two groups, including 7 patients with complete painless, 8 with mild pain, 1 with moderate pain, and 1 with severe pain in Group A. And in Group B, 7 patients had no pain, 7 had mild pain, and 1 had moderate pain. In addition, there was no statistical significance in HHS function scores of activity between the two groups.

**Table 3:** Outcome measures at last follow-up

Variable	LCP(*n* = 17)	LCP and allograft (*n* = 15)	*p*-value
HHS, mean (SD)	78.1 (16.1)	79.4 (12.9)	0.807
HHS pain, mean (SD)	39.3 (8.3)	41.2 (3.6)	0.660
HHS function, mean (SD)	31.2 (9.6)	30.9 (9.5)	0.914
Dead	1/17	1/15	1.000
Reoperation, no./total no. (%)	1/17 (0%)	2/15 (60.0%)	0.589
complications, no./total no. (%)	2/17 (0%)	2/15 (40.0%)	1.000
Rate of union, no./total no. (%)	16/17 (94.7%)	15/15 (100)	1.000
Time to union, mean (SD)	5.4 (1.6)	5.1 (1.5)	0.672
Mean follow-up (mths) (SD)	31.1 (12.8)	33.9 (15.3)	0.588

In the two groups of postoperative complications, there was a possibility of dislocation taking into account the intraoperative dislocation of the hip joint. No dislocation patients occurred in either group. One patient experienced incision infection after operation. One patient in Group B presented with persistent incision exudation, no fever, local red or other signs of infection. And the clear reddish liquid could be squeezed out during daily dressing change, but the bacterial culture was negative. One week later, incision debridement combined with intravenous antibiotics was performed, and the wound finally healed. One patient in Group B needed to be operated again due to the superficial hematoma in the incision, and the wound healed after drainage and wound compression dressing. Group A patients with nonunion fractures were transverse fractures with medial cortical discontinuity, which were fixed by Wagner SL stem (Zimmer, Warsaw IN, USA) combined with cortical allogeneic. It is worth mentioning that the patient who underwent total hip arthroplasty and subtrochanteric shorting osteotomy due to Crowe type IV Developmental dysplasia of hip (DDH) sustained a Vancouver type B1 fracture (Fig. 2a, Fig. 2b) and a standard ORIF regimen was conducted without cortical strut (Fig. 2c, Fig. 2d). As a result, she suffered from the malunion with 15°malunion at the osteotomy site (Fig. 2e) and had a poor function (HHS score 65), but no further revision was performed as economic condition (Fig. 2). There were no cases with deep vein thrombosis in the two groups during the perioperative period, and no cases with screw breakage or femoral stem loosening in the last follow-up.

## Discussion

The most important finding of this study was that LCP alone could obtain a similar clinical effect for Vancouver type B1 fracture when compared with the combined use of LCP and cortical strut allografting. A string of postoperative evaluation indexes, including the fracture union rate and HHS of patients at the last follow-up, demonstrated no statistically significant differences between the two groups. What calls for special attention is that, contrary to our findings, a previous study reported the addition of strut allograft contributed to a higher union rate [13]. We speculated that fracture union was probably related to the age of patients with fracture. The mean age of participants in Khashan et al. ‘s study was 80.4 years old while the mean age in our study was only 65.4 years old. Studies have shown that advanced age is one of the factors affecting fracture healing [22, 23], which may explain the lower union rate in the fracture group treated with the LCP alone in his study. We tried to draw firm conclusions by reviewing the previous literatures, but this remains an area of controversy. Buttaro et al. [24] treated Vancouver type B1 periprosthetic fractures with LCP alone in 9 hips, and 5 patients were treated using LCP with cortical strut allografts. Over an mean follow-up of 20 months, they reported 6 cases of nonunion, all of which occurred in 9 patients without cortical strut. Khashan et al. [13] made a similar study through retrospectively analyzing 21 cases Vancouver type B1 and C fractures. The union rate in the group treated with LCP alone was only 45.5% (5/11), while that in the group fixed by LCP and cortical strut allografts was as high as 100% (10/10). A system review of type B1 fracture by Moore et al. [25] showed that, however, there was no statistical significance in the fracture union rate between the LCP combined cortical strut group (91.5%) and the LCP group (90.7%). At the same time, the strut allograft is usually used for treat the other fractures. Rollo et al. [26] reported a superior outcome in the management of periprosthetic knee fractures without cases of nonunion using plating associated to cortical strut. Also, it’s favourable for revision in breakage of femoral nails [27]**.** The cortical strut allograft, which we believe should be effectively used when needed, such as advanced age patient, severe osteoporosis, bone defect, short transverse fracture (Fig. 3d-g) and the discontinuity of medial femoral cortex. In our study, discontinuity of the proximal femoral cortex due to subtrochanteric osteotomy in one patient didn’t get our adequate attention and the consequence is that the proximal unicortical screws fixed LCP was pulled out. We reckoned that the fixation of the proximal unicortical screws combined with cerclage wire did not appear to be stable, and that the fixation of the proximal fracture remained the weakest link in the plate structure due to the presence of the femoral stem. Moreover, gravity played an important role in malunion for some patients who were too optimistic about their condition to weight-bear prematurely. A strategy for type B1 fracture in Corten et al’s study suggested [20], the discontinuity of medial femoral cortex have been used as the indication for cortical strut. Although we have not successfully treated similar cases, the authors suggest that cortical strut fixation appears to be necessary in this condition.

On the other hand, the overall bone union time is longer than the data reported in many studies, which may be related to more extensive injury on the soft tissue, and the reason why more extensive injury is that we dislocated the patient hip in order to evaluate the stability of the prosthesis by using the posterolateral extended approach. However, the choice between minimally invasive percutaneous technique and traditional open reduction is controversial. Ricci et al. [28] obtained excellent clinical outcome by using indirect minimally invasive reduction and fixation, with a union rate of 100% in 50 Vancouver type B1 fractures and an mean union time of 12 weeks. While minimally invasive osteosynthesis has many advantages, we cannot confirm the stability of the prosthesis by means of minimally invasive percutaneous technique during the operation. Furthermore, it is still possible to misdiagnose B2 fracture as B1 fracture (e. g. Figure 3a and b). Preoperative diagnosis of type B1 fracture is considered as an independent risk factor for nonunion after treatment of periprosthetic fractures and many orthopedic surgeons underestimate type B1 fracture. In the study reported by Lindahl et al. [5], femoral stem loosening occurred in 70% of periprosthetic fractures following primary total hip arthroplasty, and undetected loosening occurred in 47% of patients. Research has found that 20% of the patients had loose femoral stem when the hip joint was dislocated to evaluate the stability of the prosthesis in 45 Vancouver type B1 fractures and that the union rate was 97%, but the mean union time was 6.4 months [20]. Yeo et al. [15] reported a similar study, in which they found that 15% (3/20) of patients had loose arthroplasty prosthesis and underwent revision surgery, while 17 patients with B1 fractures were treated with ORIF, with a union rate of 88% and an mean union time of 20 weeks. We figured that it is undoubtedly necessary to confirm the stability of prosthesis for Vancouver type B1 fracture and by traditional ORIF whenever possible. Preoperative history and radiographs alone tend to miss a portion of type B2 fracture, where intraoperative stability assessment has a significant advantage yet is associated with greater trauma. We still found 4 intraoperative B2 fractures and avoided ORIF (Fig. 3c), which is the underlying reason for the high fracture union rate in this study, and left an account of the reason for longer union time. Minimally invasive percutaneous technique in the Vancouver type B1 fractures, which benefits from a series of merits such as minimally invasive incisions, less soft tissue stripping, and biological protection of the fracture environment, improves fracture union rates and remains promising but an exactly preoperative diagnosis is indispensable. In the future, the method of definite preoperative diagnosis of B1 fracture is the next research direction.

In our series, there was one case of wound infection with persistent effusion in the Group B, but no significant difference was found in the postoperative infection rate between the two groups, which may be related to the small sample size of our study. Clinically, the fracture incidence of uncemented femoral stem is more common than that of cemented stem, but interestingly, the proportion of periprosthetic fractures after revision of the femoral stem was only 6.3%, while the figure reported by Yeo was 47.1%, Lindahl reported 28.3%. However, the data from many research reports in China is generally low. Li et al. [29] reported 39 cases of type B3 fractures, and revision accounted for only 12.8%, while in 97 cases of type B fractures reported by Zheng et al. [30], revision still accounted for only 15.5%. The reasons for these disparate results are unknown, and thus further examinations are required to warrant the current results.

There are Several limitations to the current study. First, this retrospective study design with a low level of evidence and a relatively short follow-up period. Second, our sample size is still small, which might be a possible reason for the difference between the results of our study and those of other studies. Third, the diversity of fracture types in our study led to the use of different LCPs, which to some extent influenced the allogeneic cortical strut advantage. Fourth, our study did not perform a rigorous design and different participant characteristics might diminish its accuracy.

## Conclusion

This study found that, with or without combining cortical strut allografting, ORIF followed by LCP resulted in similar fracture union rates for treatment of Vancouver type B1 and C. However, we noted that LCP alone were more likely to fail in B1 fractures type which is located in the proximal medial cortex of the femur. Therefore, we recommend the routine use of cortical strut during ORIF of Vancouver type B1 fracture with proximal medial femoral fracture. Further studies will require a larger sample size, longer-term follow-up and rigorous experimental design to determine the use of cortical strut allograft.

## Data Availability

The datasets generated and/or analysed during the current study are not publicly available due to limitations of ethical approval involving the patient data and anonymity but are available from the first author (Hui lv) on reasonable request.
